# Significance of the measurement of serum fructosamine in the management of childhood diabetes

**DOI:** 10.1186/1687-9856-2013-S1-P36

**Published:** 2013-10-03

**Authors:** Youn Shim Shin, Jiyun Park, Dong Soo Kang, Jeesuk Yu

**Affiliations:** 1 Department of Pediatrics, Dankook University Hospital, Cheonan, Republic of Korea

## Aims

HbA1c can usually be used as the indicator of glucose control and correlated with the development of long-term diabetic complications. But there are limits that it usually reflects the mean glucose levels of 2-3 months and can be variable in the situation of hemoglobinopathy or the conditions of altered RBC lifespan. In contrast, serum fructosamine levels reflect the mean glucose levels of 2-3 weeks. This study was designed to see the significance of the measurement of serum fructosamine in the management of childhood diabetes and to see the correlation between the HbA1c and fructosamine.

## Methods

Clinical data were evaluated for the sixty Korean patients who are on the management of diabetes in the department of Pediatrics, Dankook University Hospital. Fructosamine and HbA1c levels were also reviewed on the basis of clinical information and analyzed using IBM SPSS Statistics version 20.

## Results

HbA1c and fructosamine levels showed strong association (p<0.001). Fructosamine levels indicated the average glucose concentration over the previous 2-3 weeks better than HbA1c, so were useful for the evaluation of the therapeutic efficacy of recent change of therapeutic modality as well as for the diagnosis of fulminant diabetes.

## Conclusion

The measurement of fructosamine levels was useful in the management of childhood diabetes especially, if there is some discrepancy between the clinical information and HbA1 levels. In addition, it was useful for the short-term evaluation about the recent glucose control after the change of the treatment modality of diabetes.

